# Associations of genetic polymorphisms in pTEN/AKT/mTOR signaling pathway genes with cancer risk: A meta-analysis in Asian population

**DOI:** 10.1038/s41598-017-17250-z

**Published:** 2017-12-19

**Authors:** Zhen Zhang, Qiuchen Chen, Jing Zhang, Yilin Wang, Xiaoyun Hu, Sainan Yin, Miao He, Shu Guan, Wenyan Qin, Qinghuan Xiao, Haishan Zhao, Weifan Yao, Huizhe Wu, Minjie Wei

**Affiliations:** 1 Department of Pharmacology, School of Pharmacy, Liaoning Key Laboratory of Molecular Targeted Anti-Tumor Drug Development and Evaluation, China Medical University, Shenyang, 110122 P. R. China; 2 Department of Breast Surgery, First Hospital of China Medical University, Shenyang, 110001 P. R. China; 3 Department of Ion Channel Pharmacology, School of Pharmacy, China Medical University, Shenyang, 110122 P.R. China

## Abstract

The pTEN/AKT/mTOR signaling pathways play a critical role in balancing cell proliferation, differentiation, and survival. Recent studies researched the associations of core genes in the pTEN/AKT/mTOR pathway polymorphisms with the cancer susceptibility; however, the results are inconclusive. Therefore, a systematically meta-analysis was performed to evaluate the association between the five SNPs (*mTOR* rs2295080 and rs2536, *AKT1* rs2494750 and rs2494752, *pTEN* rs701848) and cancer risk by systematic review of the literature in 31 eligible studies. The results showed a significant decreased risk between rs2295080 TG, GG genotype, and GG/TG genotypes and overall cancer [TG vs.TT: OR(95% CI) = 0.82(0.76, 0.89), GG/TG vs. TT: OR(95% CI) = 0.82(0.76, 0.88), and GG vs. TG/TT: OR(95% CI) = 0.67(0.51, 0.88)] and the subgroup of urinary system cancer and digestive system cancer. Moreover, the SNP rs701848 CC, TC genotype showed significantly increased the overall cancer risk both in dominant model [CC/TC vs. TT: OR(95% CI) = 1.25(1.15, 1.36)] and recessive model [CC vs. TC/TT: OR(95% CI) = 1.20(1.09, 1.32)], and digestive system cancer and urinary system cancer. In addition, AG genotype and GG/AG genotype of rs2494752 was associated with increased risk of cancer. Therefore, this meta-analysis provided genetic risk factors for carcinogenesis and the most valid cancer prevalence estimate for Asian population.

## Introduction

Cancer is a major public health problem around the globe^1^. It is currently the second leading cause of death, and approximately 1, 658, 370 new cancer cases worldwide, 429000 new cases in China were reported according to the Cancer Statistics 2015^2^. The carcinogenesis is involved in multifactor interaction among environmental exposures, life style and internal factors. In terms of internal factors, the main manifestations are changes in hormone secretion and immune conditions, and genetic variation in the key signaling pathway. In humans, the phosphatase and tensin homolog deleted on chromosome10 (pTEN)/AKT/mammalian target of rapamycin (mTOR) signaling pathway is frequently activated in a variety of cancers, and play a critical role in many cellular processes including proliferation, differentiation, cell cycle progression, cell motility and tumorigenesis, tumor growth, angiogenesis^3–5^. Therefore, the Single nucleotide polymorphisms (SNPs) of core genes in the pTEN/AKT/mTOR pathway may impact the transcription and expression of the proteins and thus alter the capacity and function of the pathway, which could play a critical role in carcinogenesis^6–9^.

The mTOR, which is located at the chromosome 1q36.2, plays a significant role in the pTEN/AKT/mTOR pathway. It exerts a prosurvival influence on cells through the activation of factors involved in protein synthesis^10–13^. *pTEN i*s a tumor suppressor and plasma-membrane lipid phosphatase and which dephosphorylates PIP3 to PIP2, inhibiting the activation of AKT, and negatively regulates the pTEN/AKT/mTOR pathway^14^. To date, among the pTEN/AKT/mTOR pathway genes, there are more than 1000 coding-region SNPs (cSNPs) (http://www.ncbi.nlm.nih.gov/projects/SNP) reported. Among those cSNPs, a few potential functional SNPs especially located in the 5′-untranslated regions(5′UTR) and 3′UTR of the candidate genes could affect the carcinogenesis by modulating the transcriptional activity of candidate genes or by interacting with the miRNA binding, such as rs2295080 in the *mTOR* gene promoter region^15–23^, rs2494750 and rs2494752 in the *AKT1* 5′UTR region^7,15,23–27^, rs2536 in the 3′UTR of *mTOR* ^7,15–18,22,23,28–30^ and rs701848 the *pTEN* 3′UTR region^7,15,27,31–42^. Furthermore, previous studies demonstrated that the *mTOR* rs2295080 TT genotypes carriers showed a much higher mRNA levels of *mTOR* transcription by increasing the transcriptional activity of *mTOR* gene in human gastric cancer cell line SGC-7901^6^. Moreover, carrying the rs2295080 T allele showed increased mTOR mRNA levels compared with the G allele in the patients with renal cell cancer^7^ and colorectal cancer^8^. Moreover, another SNP rs2536 located in the *mTOR* 3′-UTR was predicted to affect miRNA-binding site activity. Li *et al*.^43^ found that co-transfection of the rs2536 A allele and G allele with miR-767-3p exhibited different promoter activities. Additionally, the polymorphism of *pTEN* rs701848 was proposed to involve in affecting the activity of micorRNA binding site^36^. Therefore, considering the critical role of the genetic variations in the pTEN/AKT/mTOR pathway, understanding the association between these SNPs and cancer susceptibility are urgently required.

To date, numerous studies have investigated the association of genetic polymorphisms of pTEN/AKT/mTOR pathway genes including rs2295080, rs2536 of *mTOR* gene, rs2494750 and rs2494752 in the *AKT1* gene, *pTEN* rs701848 with cancer susceptibility^6–9,15–23,28–42^, however, the results were inconclusive. Therefore, this comprehensive meta-analysis was performed in 5 SNPs of pTEN/AKT/mTOR pathway genes included all eligible case-control studies for evaluating the cancer risk and providing more precise estimation of these associations.

## Materials and Methods

### Literature research and data extraction

A comprehensive literature search was performed independently by three authors (Z.Z., J.Z., and Q.C.C.) in five electronic databases: PubMed database, CNKI, CbmWeb, WanFang Date, BIOSIS Preview, and ClinicalKey. All the searched eligible original studies and review articles were reviewed carefully to identify the relevant articles by using the following search terms “*mTOR* rs2295080” or “*mTOR* rs2536” or “*pTEN* rs701848” or “*AKT1* rs2494750” or “*AKT1* rs2494752” and “polymorphism or SNP or single nucleotide polymorphism or variation or mutation” and “cancer or carcinoma or tumor or neoplasm”, (the search was updated on Feb 15, 2017). This search was limited to these articles with English or Chinese language, and the results were reviewed and compared by a forth reviewer (Y. L.W.).

In this meta-analysis, selected publications were eligible if they fulfilled the following criteria: (1) a case-control study or cohort study design; (2) evaluated the association of the genetic polymorphisms of *mTOR*, AKT1, and *pTEN* gene with the risk of cancer; (3) sufficient genotypic and/or allelic information for estimating the odds ratio (OR) with 95% confidence intervals (CIs) was provided; (4) the samples size of cases or controls were ≥20. Animal studies, case reports, reviews, and unpublished results were excluded. The following data was extracted from each publication in the collection criterion by Z.Z. and Y. L.W. independently: first author, publication year, ethnicity, country, cancer type, control source (population-based controls, or hospital-based controls), genotyping method, the total number of genotyped cases or controls, and the number of each genotype for cases and controls with each SNP for cancer risk assessment.

### Statistical analysis

For the genotype frequency of the controls, the Hardy-Weinberg equilibrium (HWE) was assessed by using the Chi-square test or Fisher’s exact test (*P* > 0.05) in each study. The pooled odds ratios (ORs) and their corresponding 95% confidence intervals (CIs) were estimated to evaluate the strength of association between these selected 5 SNPs and cancer risk. The pooled estimated ORs and CIs were determined by *Z*-test based on homozygote model, heterozygote model, dominant model, recessive model, and an additive model(significant for *P* < 0.05). The heterogeneity between-study was assessed across all eligible comparisons by using χ^2^-based Cochran’s Q-test (significant for *P* < 0.10). The random-effects model (DerSimonian-Laird method) was chosen If there is statistical heterogeneity, otherwise the fixed-effects model (Mantel–Haenszel method) was used if the studies were homogeneous. The *I* ^2^ statistics was also determined from 0% to 100%, which quantified the heterogeneity irrespective of the number of studies. The sensitivity meta-analysis was assessed by leave-one-out each time to reflect the influence of each study to the pooled estimates. The publication bias was evaluated by the Egger’s and Begg-Matzumdar linear regression tests using asymmetry of the funnel plot (significant for *P* < 0.10). All the statistical analyses of this meta-analysis were performed by using Stata 12.0 software (StataCorp LP, College Station, USA) and Open Meta-Analyst (http://www.cebm.brown.edu/openmeta/).

## Results

### Studies extraction and characteristics

**Figure 1:**
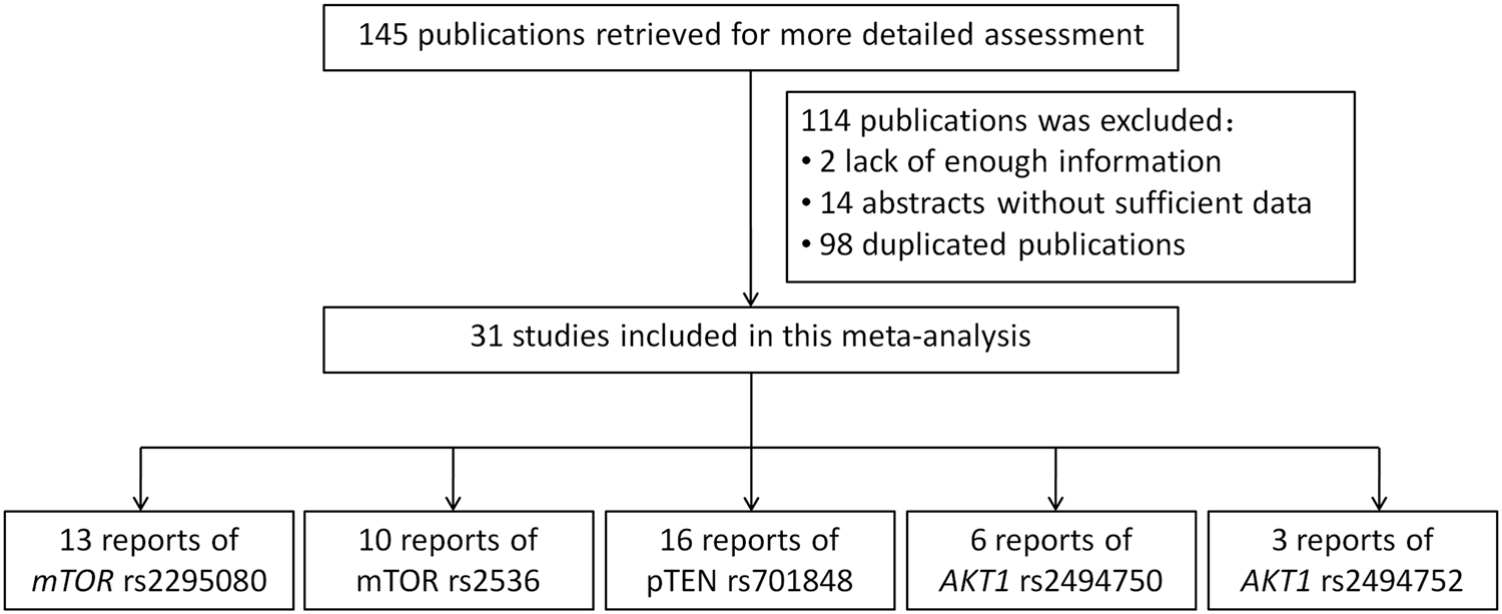
Flow of identification, inclusion, exclusion of the studies.

Figure 1 summarizes a flowchart presenting the literature review process of study identification, inclusion, exclusion. With the search strategy, a total of 145 published articles were extracted and identified for cancer risk assessment from PubMed, CNKI, CbmWeb, WanFang Date, BIOSIS Preview. After manually screening abstracts and texts of the included 145 studies, 114 were excluded for 2 lack of enough information, 14 abstracts without sufficient data, and 98 duplicated publication or overlapping with other publications for further evaluation. Finally, 31 studies of Asian population were met the inclusion criteria^6–9,15–23,28–42^, 13 studies of them evaluated the association of *mTOR* rs2295080 with cancer risks^6–9,15–23^, 10 studies determined the SNP rs2536 of *mTOR* gene and cancer susceptibility^7,15–18,22,23,28–30^, 16 publications were *pTEN* rs701848 polymorphism^7,15,27,31–42^, 6 reports studied the *AKT1* rs2494750 SNP^7,15,23–27^ and 3 reports determined *AKT1* rs2494752 SNP^19,26,27^. The distribution of genotypes in the controls for the SNPs of pTEN/AKT/mTOR pathway were in the HWE, except for these 2 publications of Liu, B *et al*.^33^ for rs70148 and Fallah *et al*.^24^ for rs2494750 (Table 1). In this final meta-analysis 8965 cases and 9868 controls for the *mTOR* rs2295080, 8411 cases and 8837 controls for the *mTOR* rs2536, 5882 cases and 6284 controls for the *pTEN* rs701848, 4332 cases and 4498 controls for the *AKT1* rs2494750, and 3187 cases and 3174 controls for the *AKT1* rs2494752 were included. The types of cancers mainly include renal cancer, prostate cancer, acute lymphoblastic leukemia (ALL), gastric cancer, hepatocellular cancer, laryngo cancer, colorectal cancer and esophageal squamous cell cancer (ESCC). The ethnicity of the included studies is Asian, and genotyping method includes TaqMan SNP genotyping assay and PCR-RFLP method. The essential characteristics for all studies were shown in Table 1.

**Table 1:** Characteristics of studies included in the meta-analysis.

Variant	Author[ref]	Year	Country	Ethnicity	Tumor type	Control Source	Genotyping method	Cases				Controls				HWE (cases)	HWE (controls)
***mTOR*** **rs2295080**								**TT**	**TG**	**GG**	**Total**	**TT**	**TG**	**GG**	**Total**		
	Cao, Q.^7^	2012	China	Asian	Renal cancer	HB	TaqMan assay	454	218	38	710	438	277	45	760	0.084	0.891
	Chen, J. W.^15^	2012	China	Asian	Prostate cancer	HB	TaqMan assay	429	209	28	666	413	259	36	708	0.690	0.573
	Huang, L.^16^	2012	China	Asian	ALL	HB	TaqMan assay	254	140	23	417	353	180	21	554	0.523	0.742
	Li, Q. X.^17^	2013	China	Asian	Prostate cancer	PB	PCR-RFLP	653	311	40	1004	617	382	52	1051	0.697	0.468
	Xu, M.^6^	2013	China	Asian	Gastric cancer	HB	PCR-RFLP	482	246	25	753	497	305	52	854	0.345	0.569
	Zhu, M. L.^18^	2015	China	Asian	ESCC	HB	TaqMan assay	674	390	49	1113	702	362	49	1113	0.432	0.788
	Xu, M.^8^	2015	China	Asian	Colorectal cancer	HB	TaqMan assay	482	225	30	737	459	273	45	777	0.563	0.602
	Wang, M. Y.^19^	2015	China	Asian	Gastric cancer	HB	TaqMan assay	568	394	40	1002	607	355	41	1003	0.005	0.221
	Zhao, P.^20^	2015	China	Asian	ALL	HB	PCR-RFLP	68	50	15	133	173	111	12	296	0.221	0.263
	Zhao, P.^20^	2015	China	Asian	AML	HB	PCR-RFLP	27	14	6	47	173	111	12	296	0.080	0.263
	Zhu, J. H.^21^	2015	China	Asian	Renal cancer	HB	TaqMan assay	674	390	49	1113	702	362	49	1113	0.432	0.788
	Zhao, Y.^22^	2016	China	Asian	Breast cancer	HB	Sequencing	351	197	12	560	345	212	26	583	0.009	0.358
	Zhang, J.^23^	2016	China	Asian	Renal cancer	HB	TaqMan assay	454	218	38	710	438	277	45	760	0.084	0.891
***mTOR*** **rs2536**								**TT**	**TC**	**CC**	**Total**	**TT**	**TC**	**CC**	**Total**		
	Cao, Q.^7^	2012	China	Asian	Renal cancer	HB	TaqMan assay	607	99	4	710	628	128	4	760	0.001	0.353
	Chen, J. W.^15^	2012	China	Asian	Prostate cancer	HB	TaqMan assay	565	96	5	666	585	119	4	708	0.697	0.435
	Huang, L.^16^	2012	China	Asian	ALL	HB	TaqMan assay	346	65	6	417	448	103	3	554	0.153	0.258
	Li, Q.^17^	2013	China	Asian	Prostate cancer	PB	PCR-RFLP	804	192	8	1004	894	147	10	1051	0.346	0.156
	Zhu, M. L.^18^	2013	China	Asian	ESCC	HB	TaqMan assay	951	165	7	1123	957	157	7	1121	0.957	0.839
	Mao, L. Q.^28^	2013	China	Asian	Hepatocellular cancer	HB	TaqMan assay	849	186	13	1048	850	188	14	1052	0.439	0.330
	He, J.^29^	2013	China	Asian	Gastric cancer	HB	TaqMan assay	938	179	8	1125	1019	170	7	1196	0.865	0.975
	Liu, Y. C.^30^	2014	China	Asian	Hepatocellular cancer	HB	TaqMan assay	849	186	13	1048	850	188	14	1052	0.439	0.330
	Zhang, J.^23^	2016	China	Asian	Renal cancer	HB	TaqMan assay	607	99	4	710	628	128	4	760	0.987	0.353
	Zhao, Y.^22^	2016	China	Asian	Breast cancer	HB	Sequencing	453	100	7	560	486	93	4	583	0.580	0.845
***pTEN*** **rs701848**								**TT**	**TC**	**CC**	**Total**	**TT**	**TC**	**CC**	**Total**		
	Zou, J. F.^31^	2006	China	Asian	Laryngo cancer	HB	PCR-RFLP	17	23	12	52	28	52	24	104	0.547	0.135
	Liu, B.^33^	2008	China	Asian	Laryngo cancer	HB	PCR-RFLP	7	20	12	91	13	22	9	104	0.578	**0.008**
	Zhai, Y.^32^	2009	China	Asian	Laryngo cancer	HB	PCR-RFLP	29	45	17	39	26	54	24	44	0.144	0.074
	Song, Z. X.^34^	2009	China	Asian	Laryngo cancer	HB	PCR-RFLP	46	74	29	149	26	54	24	104	0.791	0.073
	Shi, G. L.^35^	2009	China	Asian	Lung cancer	HB	PCR-RFLP	21	43	13	77	24	54	26	104	0.026	0.134
	Song, Z. X.^42^	2009	China	Asian	Gastric cancer	HB	PCR-RFLP	43	67	21	58	65	116	34	104	0.311	0.253
	Ding, J.^36^	2011	China	Asian	Hepatocellular cancer	HB	PCR-RFLP	222	338	150	131	277	351	132	215	0.797	0.788
	Cao, Q.^7^	2012	China	Asian	Renal cancer	HB	TaqMan assay	70	121	35	710	103	90	33	760	0.055	0.691
	Chen, J. W.^15^	2012	China	Asian	Prostate cancer	HB	TaqMan assay	212	329	125	666	235	353	120	708	0.789	0.956
	Jang, Y.^38^	2013	China	Asian	ESCC	HB	PCR-RFLP	91	155	58	304	183	165	65	413	0.950	0.692
	Tang, Q. S.^27^	2014	China	Asian	Breast cancer	HB	TaqMan assay	239	519	212	970	280	486	168	934	0.938	0.692
	Zhang, Y. G.^41^	2014	China	Asian	ESCC	HB	PCR-RFLP	205	182	38	494	243	182	21	494	0.894	0.519
	Xu, X.^39^	2015	China	Asian	ESCC	HB	TaqMan assay	186	421	173	425	229	397	138	446	0.257	0.692
	Lin, L.^40^	2015	China	Asian	Colorectal cancer	HB	TaqMan assay	222	338	150	780	277	351	132	764	0.027	0.088
	Liu, N.^37^	2015	China	Asian	ESCC	HB	PCR-RFLP	173	241	80	226	145	248	101	226	0.440	0.988
	Zhang, J.^23^	2016	China	Asian	Renal cancer	HB	TaqMan assay	17	35	6	710	24	54	26	760	0.311	0.253
***AKT1*** **rs2494750**								**GG**	**GC**	**CC**	**Total**	**GG**	**GC**	**CC**	**Total**		
	Cao, Q.^7^	2012	China	Asian	Renal cancer	HB	TaqMan assay	300	340	70	710	349	328	83	760	0.062	0.652
	Chen, J. W.^15^	2012	China	Asian	Prostate cancer	HB	TaqMan assay	80	269	317	666	78	299	331	708	0.053	0.399
	Fallah, S^24^	2015	Iran	Asian	Endometrial cancer	HB	PCR-RFLP	19	6	5	30	22	5	3	30	0.007	0.015
	Zhang, J.^23^	2016	China	Asian	Renal cancer	HB	TaqMan assay	300	340	70	710	349	328	83	760	0.062	0.652
	Wang, M. Y.^25^	2016	China	Asian	Gastric cancer	HB	TaqMan assay	493	480	126	1099	545	487	112	1144	0.577	0.833
	Zhu, J. H.^26^	2016	China	Asian	ESCC	HB	TaqMan assay	555	448	114	1117	521	460	115	1096	0.098	0.371
***AKT1*** **rs2494752**								**AA**	**AG**	**GG**	**Total**	**AA**	**AG**	**GG**	**Total**		
	Tang, Q. S.^27^	2014	China	Asian	Breast cancer	HB	TaqMan assay	300	511	159	970	331	464	139	934	0.017	0.253
	Wang, M. Y.^25^	2016	China	Asian	Gastric cancer	HB	TaqMan assay	547	454	99	1100	623	430	91	1144	0.730	0.167
	Zhu, J. H.^26^	2016	China	Asian	ESCC	HB	TaqMan assay	611	423	83	1117	597	415	84	1096	0.409	0.317

HB, Hospital based; PB, Population based; PCR-RFLP, polymorphism chain reaction- restriction fragment length polymorphism; ALL, Acute lymphocytic leukemia; ESCC, Esophageal squamous cell carcinoma; AML, Acute myeloid leukemia.

### Meta-analysis results

#### mTOR rs2295080, rs2536 and cancer risk analysis

**Figure 2:**
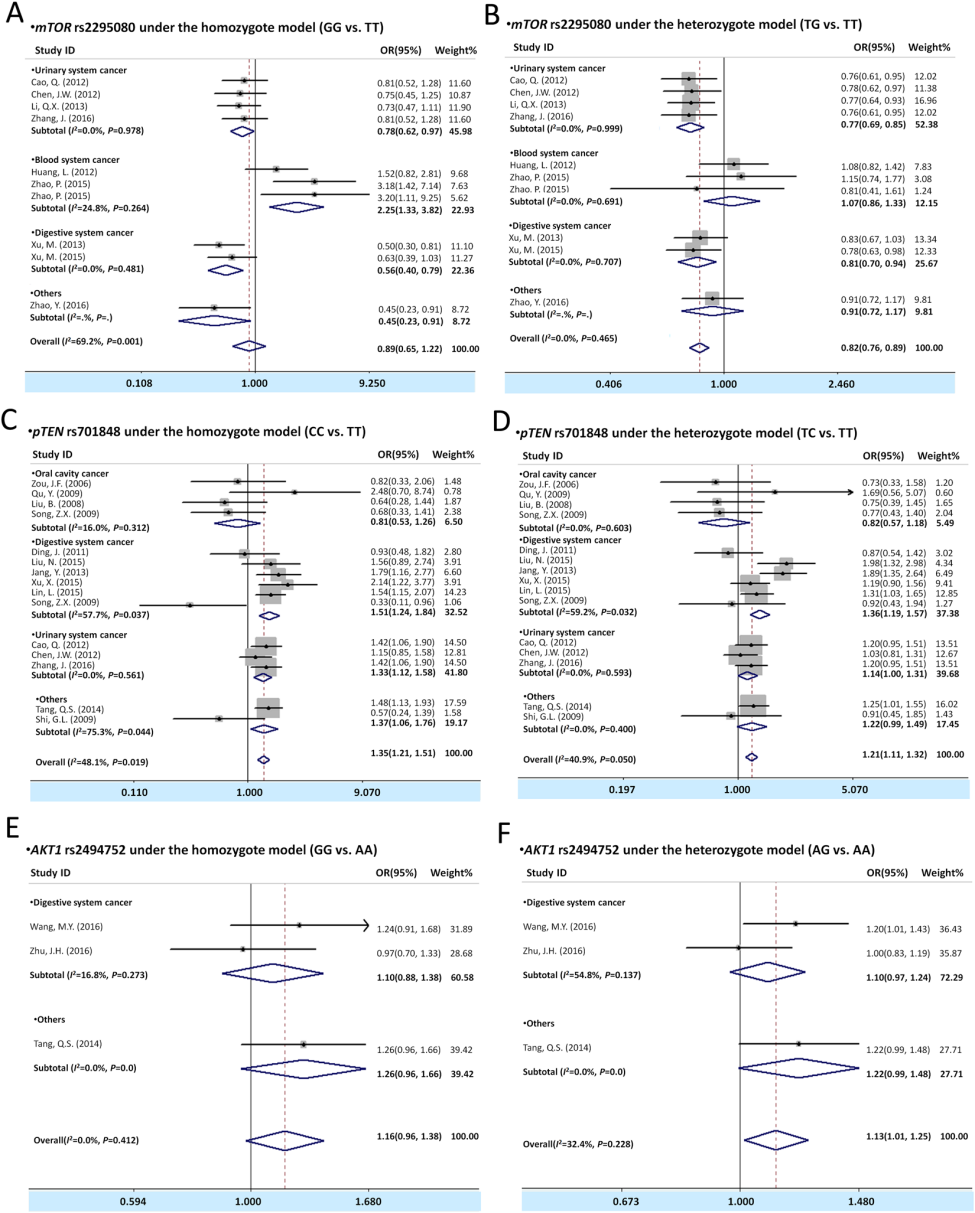
Forest plots of cancer risk with polymorphism of *mTOR* rs2529080 (**A,B**), *pTEN* rs701848 (**C,D**) and *AKT1* rs2494752 (**E,F**) assessing by subgroup analysis under the homozygoute model (**A,C,E**), heterozygote model (**B,D,F**). The estimates of OR(95% CIs) are plotted with a box and a horizontal line for each study. ◇, pooled ORs (95% CIs).

The meta-analysis results for the *mTOR* rs2295080 and rs2536 polymorphism and cancer susceptibility are illustrated in Tables 2, 3, Fig. 2, and Figure S1. Overall, we observed that carrying *mTOR* rs2295080 TG or GG genotype and GG/TG genotype showed significant association with decreased cancer risk [TG vs.TT in heterozygote model: OR(95% CI) = 0.82(0.76, 0.89), *P* < 0.001; GG/TG vs. TT in dominant model: OR(95% CI) = 0.82(0.76, 0.88), *P* < 0.001; and GG vs.TG/TT in recessive model: OR(95% CI) = 0.67(0.51, 0.88), *P* = 0.004]. In view of the relative higher heterogeneities, we further analyzed the data by stratification subgroups of urinary system cancer, blood system cancer, and digestive system cancer. Subsequently, we found that rs2295080 GG genotype, TG genotype, and GG/TG genotypes carriers showed a significantly decreased cancer risk in the stratification analysis of urinary system cancer [GG vs.TT: OR(95% CI) = 0.78(0.62, 0.97), *P* = 0.029; TG vs. TT: OR(95% CI) = 0.77(0.69, 0.85), *P* < 0.001; GG/TG vs. TT: OR(95% CI) = 0.77(0.69, 0.85), *P* < 0.001; and GG vs. TG/TT: OR(95% CI) = 0.79(0.63, 0.98), *P* = 0.035] and digestive system cancer [GG vs.TT: OR(95% CI) = 0.56(0.40, 0.79), *P* = 0.001; TG vs. TT: OR(95% CI) = 0.81(0.70, 0.94), *P* = 0.006; GG/TG vs. TT: OR(95% CI) = 0.77(0.67, 0.89), *P* = 0.001; and GG vs. TG/TT: OR(95% CI) = 0.55(0.40, 0.77), *P* = 0.001]. However, in the subgroup analysis of blood system cancer, inversely results were found that a significantly increased cancer risk was observed in the carriers of GG genotype [GG vs.TT: OR(95% CI) = 2.25(1.33, 3.82), *P* = 0.003]. For *mTOR* rs2536 polymorphism, there was no association was observed both in overall analysis and subgroup analysis (Tables 2 and 3).

**Table 2:** Meta-analysis of the association between genetic polymorphisms of PTEN/AKT/mTOR pathway and cancer risk.

Variables	No. of cases/controls	*P* ^z*^	Homozygous OR(95% CI)	*P* ^het#^	*I* ^2#^ (%)	*P* ^z^*	Heterozygous OR(95% CI)	*P* ^het#^	*I* ^2#^ (%)
***mTOR*** **rs2295080**			**GG vs. TT**				**TG vs. TT**		
Urinary system cancer^†^	4203/4392	**0.029**	**0.78(0.62, 0.97)**	0.978	0.0	**0.000**	**0.77(0.69, 0.85)**	0.999	0.0
Blood system cancer^§^	5971/1146	**0.003**	**2.25(1.33, 3.82)**	0.264	24.8	0.574	1.07(0.86, 1.33)	0.691	0.0
Digestive system cancer^¶^	3605/3747	**0.001**	**0.56(0.40, 0.79)**	0.481	0.0	**0.006**	**0.81(0.70, 0.94)**	0.707	0.0
**Overall**	8965/9868	0.456	0.89(0.65, 1.22)	0.001	69.2	**0.000**	**0.82(0.76, 0.89)**	0.465	0.0
***mTOR*** **rs2536**			**CC vs. TT**				**TC vs. TT**		
Urinary system cancer	3090/3279	0.954	1.02(0.56, 1.86)	0.976	0.0	0.729	0.95(0.69, 1.29)	0.001	81.0
Digestive system cancer	417/554	0.975	0.99(0.64, 1.53)	0.97	0.0	0.473	1.04(0.93, 1.17)	0.789	0.0
Blood system cancer	4344/4421	0.181	2.59(0.64, 10.43)	—	—	0.245	0.82(0.58, 1.15)	—	—
**Overall**	8411/8837	0.555	1.10(0.80, 1.53)	0.968	0.0	0.998	1.00(0.89, 1.13)	0.022	53.7
***pTEN*** **rs701848**			**CC vs. TT**				**TC vs. TT**		
Oral cavity cancer^¥^	331/356	0.359	0.81(0.53, 1.26)	0.312	16.0	0.292	0.82(0.57, 1.18)	0.603	0.0
Digestive system cancer	2418/2662	**0.000**	**1.51(1.24, 1.84)**	0.037	57.7	**0.000**	**1.36(1.19, 1.57)**	0.032	59.2
Urinary system cancer	2086/2228	**0.001**	**1.33(1.12, 1.58)**	0.561	0.0	**0.049**	**1.14(1.00, 1.31)**	0.539	0.0
**Overall**	5882/6284	**0.000**	**1.35(1.21, 1.51)**	0.019	48.1	**0.000**	**1.21(1.11, 1.32)**	0.050	40.9
***AKT1*** **rs2494750**			**CC vs. GG**				**GC vs. GG**		
Urinary system cancer	2086/2228	0.830	0.97(0.76, 1.24)	0.671	0.0	0.050	3.60(1.00, 12.97)	0.000	96.0
Reproductive system cancer^$^	30/30	0.621	0.93(0.70, 1.24)	—	—	0	0.01(0.01, 0.03)	—	—
Digestive system cancer	2216/2240	0.264	1.13(0.91, 1.41)	0.304	5.3	0.292	1.31(0.79, 2.17)	0.000	97.5
**Overall**	4332/4498	0.727	1.03(0.89, 1.18)	0.658	0.0	0.943	1.03(0.49, 2.17)	0.000	97.5
***AKT1*** **rs2494752**			**GG vs. AA**				**AG vs. AA**		
Digestive system cancer	2217/2240	0.397	1.10(0.88, 1.38)	0.273	16.8	0.151	1.10(0.97, 1.24)	0.137	54.8
Other cancer	970/934	0.098	1.26(0.96, 1.66)	—	—	0.057	1.22 (0.99, 1.48)	—	—
**Overall**	3187/3174	0.090	1.16(0.96, 1.38)	0.412	0.0	**0.026**	**1.13(1.01, 1.25)**	0.228	32.4

^*^ *P* ^z^: the significance of the pooled OR was determined by Z-test, and *P* < 0.05 was considered as statistically significant.

^#^ *P* ^het^ and *I* ^2^ were calculated by Chi square-based Q-test.

^†^Urinary system cancer: renal cancer, prostate cancer; ^§^Blood system cancer: acute lymphocytic leukemia, acute myeloid leukemia; ^¶^Digestive system cancer: gastric cancer, ESCC, hepatocellular cancer, colorectal cancer; ^¥^Oral cavity cancer: laryngo cancer; ^$^Reproductive system cancer: endometrial cancer.

**Table 3:** Meta-analysis of the association between genetic polymorphisms of PTEN/AKT/mTOR pathway and cancer risk by recessive and dominant models.

Variables	*P* ^z*^	Dominant OR(95% CI)	*P* ^het#^	*I* ^2#^ (%)	*P* ^z*^	Recessive OR(95% CI)	*P* ^het#^	*I* ^2#^ (%)
***mTOR*** **rs2295080**		**GG/TG vs. TT**				**GG vs. TG/TT**		
Urinary system cancer^†^	**0.000**	**0.77(0.69, 0.85)**	1.000	0.0	**0.035**	0.79(0.63, 0.98)	0.827	0.0
Blood system cancer^§^	0.142	1.17(0.95, 1.44)	0.722	0.0	0.742	0.91(0.52, 1.59)	0.139	49.3
Digestive system cancer^¶^	0.001	0.77(0.67, 0.89)	0.867	0.0	**0.001**	0.55(0.40, 0.77)	0.809	0.0
**Overall**	**0.000**	**0.82(0.76, 0.88)**	0.113	36.9	**0.004**	0.67(0.51, 0.88)	0.004	67.2
*mTOR* rs2536		CC/TC vs. TT				CC vs. TC/TT		
Urinary system cancer	0.836	0.99(0.86, 1.13)	0.002	79.3	0.972	1.01(0.55, 1.85)	0.952	0.0
Digestive system cancer	0.489	1.04(0.93, 1.16)	0.749	0.0	0.960	0.99(0.64, 1.53)	0.976	0.0
Blood system cancer	0.400	0.87(0.62, 1.21)	—	—	0.165	2.68(0.67, 10.78)	—	—
**Overall**	0.649	1.02(0.94, 1.10)	0.036	49.7	0.572	1.10(0.79, 1.53)	0.961	0.0
***pTEN*** **rs701848**		**CC/TC vs. TT**				**CC vs. TC/TT**		
Oral cavity cancer^¥^	0.674	0.82(0.58, 1.15)	0.568	0.0	0.250	0.92(0.64, 1.34)	0.409	0.0
Digestive system cancer	**0.017**	1.40(1.22, 1.59)	0.061	52.5	**0.000**	**1.23(1.04, 1.47)**	0.027	60.4
Urinary system cancer	**0.008**	1.20(1.05, 1.36)	0.784	0.0	**0.006**	**1.23(1.05, 1.43)**	0.476	0.0
**Overall**	**0.000**	1.25(1.15, 1.36)	0.179	24.9	**0.000**	**1.20(1.09, 1.32)**	0.015	49.9
***AKT1*** **rs2494750**		**CC/GC vs. GG**				**CC vs. GC/GG**		
Urinary system cancer	0.310	1.09(0.92, 1.30)	0.369	0.0	0.999	1.00(0.84, 1.20)	0.575	0.0
Reproductive system cancer^$^	0.312	0.92(0.78, 1.08)	—	—	0.825	0.97(0.74, 1.28)	—	—
Digestive system cancer	0.055	1.13(1.00, 1.29)	0.784	0.0	0.557	1.07(0.86, 1.31)	0.185	43
**Overall**	0.198	1.06(0.97, 1.16)	0.293	18.5	0.810	1.02(0.90, 1.15)	0.67	0.0
***AKT1*** **rs2494752**		**GG/AG vs. AA**				**GG vs. AG/AA**		
Digestive system cancer	0.129	1.10(0.97, 1.23)	0.098	63.5	0.614	1.06(0.85, 1.31)	0.447	0.0
Other cancer	0.037	1.23(1.01, 1.48)	—	—	0.365	1.12(0.88, 1.44)	—	—
**Overall**	0.017	1.13(1.02, 1.25)	0.157	46.0	0.329	1.08(0.92, 1.28)	0.704	0.0

^*^ *P* ^z^: the significance of the pooled OR was determined by *Z*-test, and *P* < 0.05 was considered as statistically significant.

^#^ *P* ^het^ and *I* ^2^ were calculated by Chi square-based Q-test.

^†^Urinary system cancer: renal cancer, prostate cancer; ^§^Blood system cancer: acute lymphocytic leukemia, acute myeloid leukemia; ^¶^Digestive system cancer: gastric cancer, ESCC, hepatocellular cancer, colorectal cancer; ^¥^Oral cavity cancer: laryngo cancer; ^$^Reproductive system cancer: endometrial cancer.

#### pTEN rs701848 and cancer risk analysis

**Figure 3:**
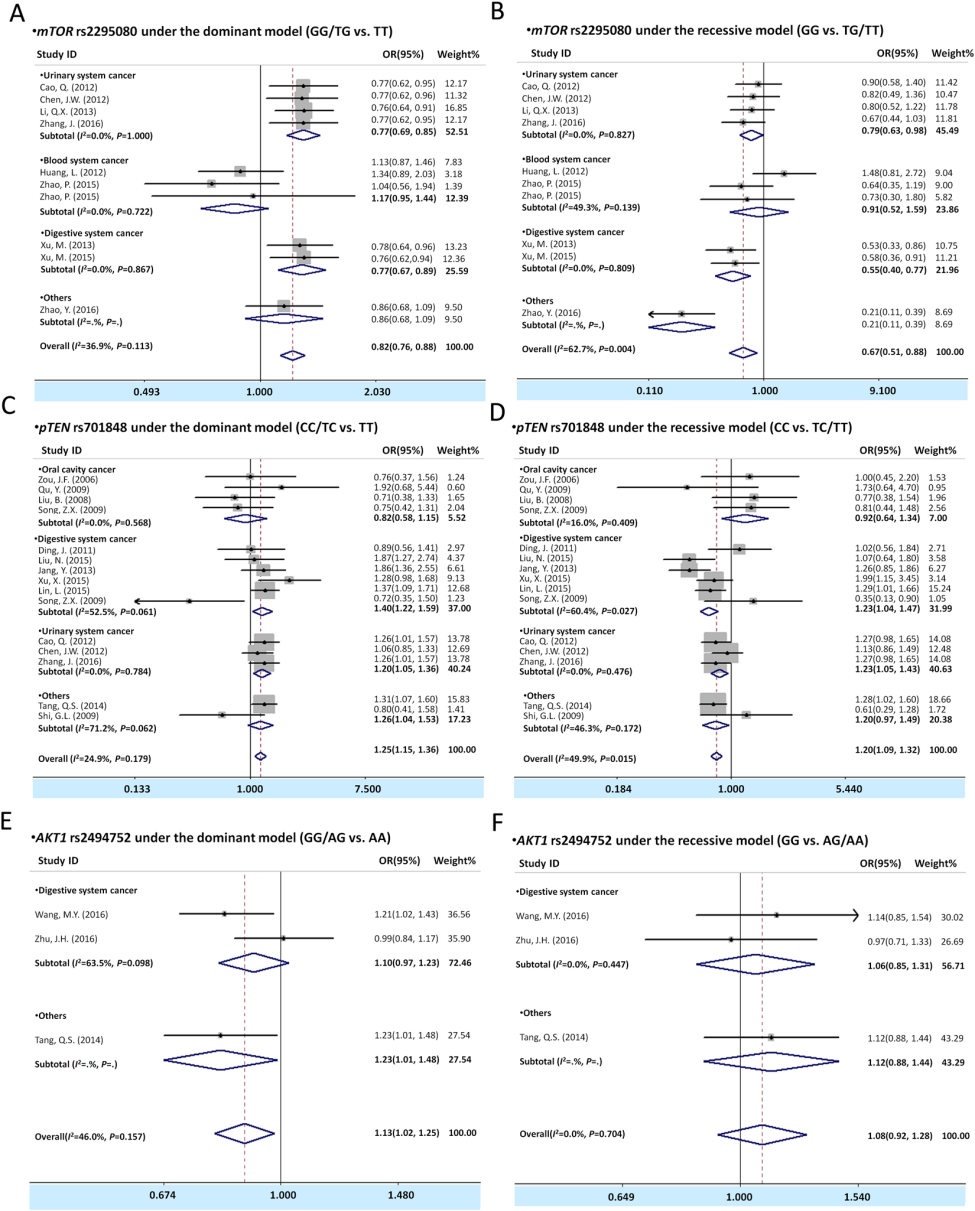
Forest plots of cancer risk with polymorphism of *mTOR* rs2529080 (**A,B**), *pTEN* rs701848 (**C,D**) and *AKT1* rs2494752 (**E,F**) assessing by subgroup analysis under the dominant model (**A,C,E**) and recessive model (**B,D,F**). The estimates of OR(95% CIs) are plotted with a box and a horizontal line for each study. ◇, pooled ORs (95% CIs).

The effect of *pTEN* rs701848 polymorphism on cancer risk in overall and subgroup analysis was shown in Tables 2, 3 and Figs 3 and S2.The SNP rs701848 CC or TC genotype and CC/TC genotype were associated with an increased overall cancer risk [CC vs.TT in homozygote model: OR(95% CI) = 1.35(1.21, 1.51), *P* < 0.001; TC vs. TT in heterozygote model: OR(95% CI) = 1.21(1.11, 1.32), *P* < 0.001; CC/TC vs. TT in dominant model: OR(95% CI) = 1.25(1.15, 1.36), *P* < 0.001; and CC vs. TC/TT in recessive model: OR(95% CI) = 1.20(1.09, 1.32), *P* < 0.001]. In the further stratification analysis, we found that rs701848 CC genotype, TC genotype and CC/TC genotypes were statistically increased association with digestive system cancer [CC vs.TT: OR(95% CI) = 1.51(1.24, 1.84), *P* < 0.001; TC vs. TT: OR(95% CI) = 1.36(1.19, 1.57), *P* < 0.001; and CC/TC vs. TT: OR(95% CI) = 1.40(1.22, 1.59), *P* = 0.017; and CC vs. TC/TT: OR(95% CI) = 1.23(1.04, 1.47), *P* < 0.001] and urinary system cancer[CC vs. TT: OR(95% CI) = 1.33(1.12, 1.58), *P* < 0.001; TC vs. TT: OR(95% CI) = 1.14(1.00, 1.31), *P* = 0.049; and CC/TC vs. TT: OR(95% CI) = 1.20(1.05, 1.36), *P* = 0.008; and CC vs. TC/TT: OR(95% CI) = 1.23(1.05, 1.43), *P* = 0.006], however no association was observed between the oral cavity cancer and cancer risk in this concluded studies.

### AKT1 rs2494750, rs2494752 and cancer risk analysis

The association between polymorphisms of *AKT1* rs2494750, rs2494752 and cancer risk in overall meta-analysis results was shown in Tables 2, 3 and Fig. 3, and Figure S2. A significant association was observed between *AKT1* rs2494752 and overall cancer risk, and the heterozygous genotype AG and GG/AG genotype of *AKT1* rs2494752 were associated with increased cancer risk (AG vs. AA in heterozygote model: OR(95% CI) = 1.13(1.01, 1.25), *P* = 0.026; and GG/AG vs. AA in dominant model: OR(95% CI) = 1.13(1.02, 1.25), *P* = 0.017). For *AKT1* rs2494750 polymorphism, we have not found the correlation with the cancer susceptibility both in overall analysis and subgroup analysis (Tables 2 and 3).

### Heterogeneity and sensitivity analysis

No significant heterogeneities were observed for the overall analyses of *mTOR* rs2536, *AKT1* rs2494750 and rs2494752. However, the highest heterogeneity were observed when all the studies were analyzed for all the cases of *mTOR* rs2295080 under the dominant model (*I* ^2^ = 96.1%) and *pTEN* rs701848 under the dominant model (*I* ^2^ = 62.0%) (Tables S1 and S2). The heterogeneity of *mTOR* rs2295080 was also present in the subgroup of urinary system cancer and digestive system cancer under the dominant model (Table S2). Therefore, the leave-one-out sensitivity analysis and random-effects model was selected for decreasing the heterogeneity. When these 3 publications of Zhu, M. L. (2015), Wang, M.Y. (2015), Zhu, J.H. (2015) were deleted, the value of *I* ^2^ decreased from 96.1% to 39.6% under the dominant model. More importantly, after deleting these 2 articles of Zhu, M. L. (2015), Wang, M.Y. (2015) from digestive system cancer, the subgroup of heterogeneity significantly decreased from 97.6% to 0% (Tables 3 and S2). Another article of Zhu, J.H. (2015) from urinary system cancer was deleted, the *I* ^2^ decreased from 96.3% to 0% under the dominant model in subgroup analysis (Tables 3 and S2). For *pTEN* rs701848, we found that the heterogeneity of overall and subgroup was significantly decreased after deleting this article of Zhang Y.G.(2014), except the subgroup under the digestive system cancer (Tables 2, 3, S1 and S2).

### Publication bias analysis

**Figure 4:**
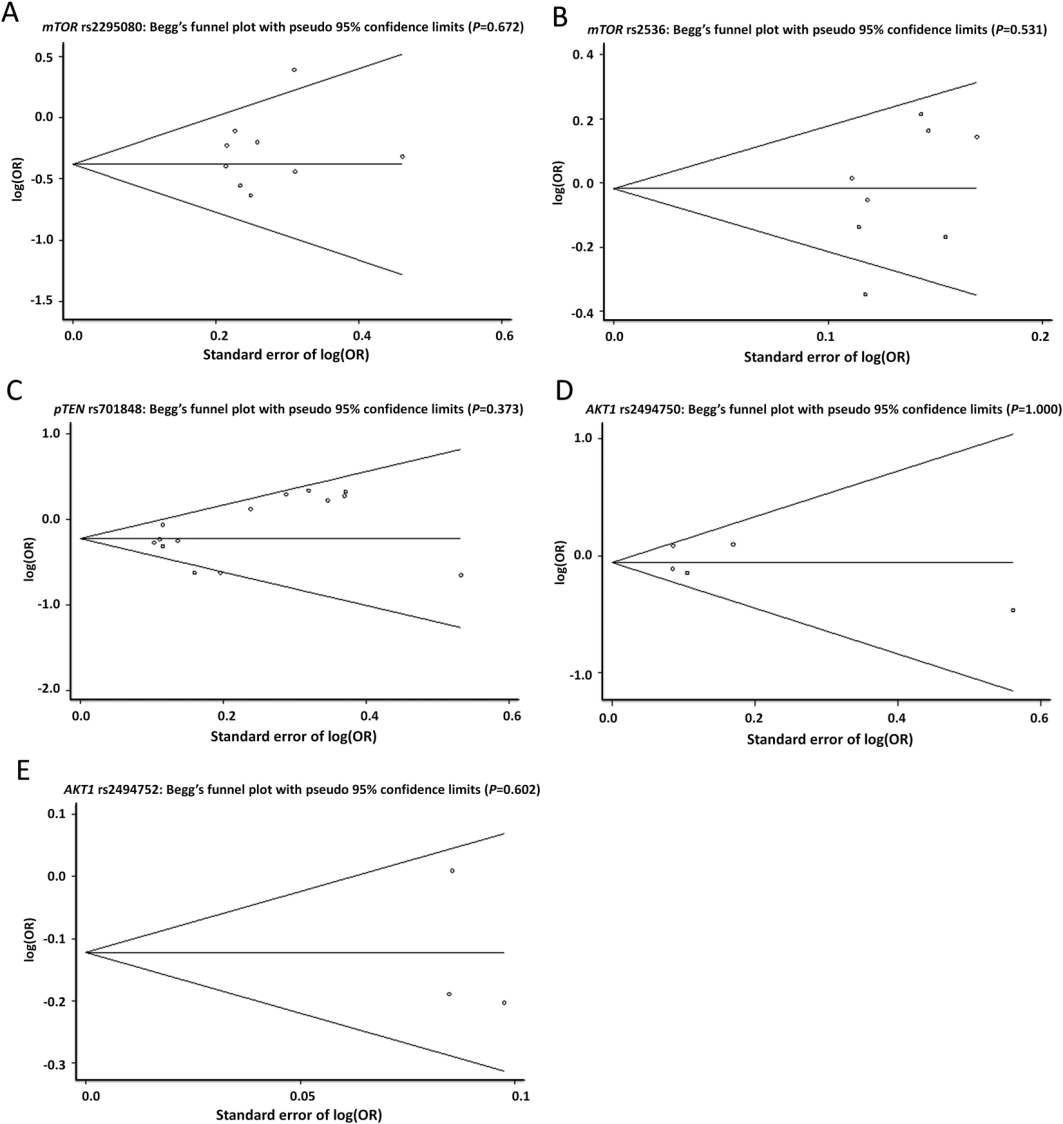
Begg’s funnel plots to detect publication bias with pseudo 95% CIs under the dominant model. Each point represents an independent study for the indicated association. (**A**) *mTOR* rs2295080; (**B**) *mTOR* rs2536; (**C**) *pTEN* rs701848; (**D** and **E**) *AKT1* rs2494750 and rs2494752.

In this meta-analysis, the Begg’s funnel plot and Egger’s test were performed to evaluate the publication bias of the concluded studies. There are no significant publication bias was observed for all the dominant models of the five SNPs (rs2295080: *P* = 0.200; rs2536: *P* = 0.176; rs701848: *P* = 0.218; rs2494750: *P* = 0.694 and rs2494752: *P* = 0.696) by the Egger’s test. The funnel plots shapes showed obvious symmetry, which were obtained for the association of the SNPs under the dominant model (rs2295080: *P* = 0.672; rs2536: *P* = 0.531; rs701848: *P* = 0.373; rs2494750: *P* = 1.000 and rs2494752: *P* = 602) (Fig. 4). The data indicated that no publication bias might have a significant influence on the observed effect of SNPs located at pTEN/AKT/mTOR pathway on the susceptibility of cancer as assessed.

## Discussion

Overexpression or mutations of key genes of the pTEN/AKT/mTOR pathways were associated with carcinogenesis, invasion, metastasis, and prognosis of a variety of cancers^44,45^. Since one group investigated the association of genetic polymorphisms of the pTEN/AKT/mTOR pathway with cancer risk for the first time^31^, a variety of studies have been performed to explore the possible correlation of the SNPs in this pathway genes on cancer susceptibility^6–9,15–23,28–42^. The potentially functional SNPs in those key genes, especially in the TFBS or miRNA binding sites may involve in the cancer susceptibility. Therefore, the present meta-analysis analyzed the associations of SNPs in the 5′upstream regulatory or promoter region (*mTOR* rs2295080, *AKT1* rs2494752), and 3′UTR region (*mTOR* rs2536, *pTEN* rs701848 and *AKT1* rs2494750) in the mTOR signaling pathway (*AKT*, and *PTEN*) with cancer risk.

### mTOR rs2295080 and rs2536 SNPs and cancer risk

Given the crucial function of *mTOR* in cellular signals from growth factors and energy status, such as in angiogenesis and cell proliferation, growth, differentiation, and apoptosis^3–5,44,45^, our findings of an association between the genetic variations in *mTOR* gene and cancer risk are biologically plausible and wide, including renal cell cancer^7,21,23^, prostate cancer^15,17^, breast cancer^22^, acute lymphocytic leukemia^16,20^, gastric cancer^19,29^, esophageal squamous cell cancer^18^, hepatocellular cancer^23,28^ and colorectal cancer^8^. In this meta-analysis of 13 studies including 8965 cases and 9868 controls for rs2295080, we found a significant decreased of rs2295080 TG, GG genotype, G allele and TG/GG genotype on the overall cancer risk, and the stratification subtype of urinary system cancer and digestive system cancer. However, a significant increased association was found on the blood system cancer in the homozygous GG genotype and G allele under the subgroup analysis. Up to now, only two meta-analyses focused on *mTOR* rs2295080 polymorphism and cancer risk^46,47^. In one meta-analysis reported that the rs2295080 G allele is associated with decreased risk of cancer^46^, however, only five studies were included. Recent some opposite findings were reported in gastric cancer^6^, esophageal cancer^18^, and especially in acute leukemia^16,20^. Thereafter, another eight studies meta-analysis found that the rs2295080 G allele was associated with a significantly lower risk of genitourinary cancers in the dominant model, and a higher risk of acute leukemia in the recessive model^47^, which consistent with our founding’s in overall cancer risk and digestive system cancer. Likewise, we further founded that carrying rs2295080 GG genotype showed increased 2.25-fold association in the blood system cancer including acute lymphocytic leukemia and acute lymphocytic leukemia which was not explicated in the previous meta-analyses. Thus, these data indicated that there was an obvious divergence between the SNP rs2295080 and cancer risk in the digestive system and blood system, which might be partially explained by cancer-specificity of rs2295080 polymorphism.

10 studied of 8411 cases and 8837 controls for rs2536 polymorphism, no significant association was observed between rs2536 and cancer susceptibility after performing stratified analyses by cancer type^7,15–18,22,23,28–30^ in this pooled meta-analysis. Previously, Shao *et al*.^46^ performed a six case-control studies meta-analysis and also reported that there was no association of rs2536 SNP with cancer risk both under dominant and recessive models. Furthermore, this present updated meta-analysis also indicated that rs2536 polymorphism was not an important biomarker for predicting cancer risk, although rs2536 was observed associated with the risk of esophageal cancer^18^ and prostate cancer^15^, together with interacting with environmental factors such as body mass index. The previous foundlings were controversial for the SNP rs2536, partially because of insufficient statistical power and further studies of different cancers are needed for providing a more precise estimation of the associations.

### pTEN rs701848 and cancer risk

*pTEN* was originally identified as tumor suppressor gene, considered as a key negative regulator of PI3K/Akt pathway^48–50^. However, little is known about the association between *pTEN* rs701848 polymorphism and cancer. Specifically, 15 studies of 5882 cases and 6284 controls for rs701848, CC or TC genotype, C allele and CC/TC genotype were associated with significant increased overall cancer risk and in the subgroup of the digestive system cancer and urinary system cancer, but not in oral cavity cancers. Since Zou *et al*.^31^ for the first time reported a significant association of *pTEN* rs701848 with laryngo cancer risk in 2006, more evidences have accumulated regarding the relationship between SNP rs701848 and the risk of various cancers, such as lung cancer^35^, esophageal cancer^37–39,41^, breast cancer^27^, prostate cancer^15^, hepatocellular cancer^36^, renal cancer^7,23^, gastric cancer^42^, and colorectal cancer^40^. It should be noted that SNP rs701848 is located within the 3′ near gene region, which can be targeted by microRNAs to affecting the miRNA binding site activity, thereby altering pTEN expression by influencing the mRNA stability, and then influence cancer susceptibility. However, the hypothesized function about SNP rs701848 still needs to be investigated in future studies and updated meta-analysis.

### AKT1 rs2494750, rs2494752 and cancer risk

In this pooled meta-analysis, 6 studied of 4332 cases and 4498 controls for rs2494750 polymorphism^7,15,23–27^ and 3 studied of 3187 cases and 3174 controls for rs2494752 polymorphism^19,26,27^ were included. A significant association was observed between rs2494752 and overall cancer risk, the heterozygous genotype AG, GG/AG genotype and G allele of rs2494752 SNP was associated with increased cancer risk. However, we have not found the correlation between rs2494750 polymorphism and the cancer susceptibility both in overall analysis and the stratification analysis. Previous study reported^15^ that the risk effect of rs2494752 AG/GG genotypes was more obvious in the patients of ever-drinkers. Another investigation showed inconsistent results in alcohol consumption patients and risk of gastric cancer^25^. These results indicated that the environmental factors were interacted with the genetic variants in the aspect of carcinogenesis. Most importantly, the SNP rs2494752 is located at the 5′ UTR of *AKT1* gene, a region predicted to be the transcription factor binding region based on the sequence alignments, which may affect the transcription and translation of *AKT1*. It is plausible that the rs2494752 G allele increased the transcription activity of the promoter in the *AKT1* gene, then facilitated the cancer development and progression, which may partially explain the cancer risk associated with this SNP. However, the potential function of this SNP should be necessary to identify in larger sample studies in the future.

There is not previously reported meta-analysis to date that comprehensively elucidated the association between the five SNPs of the pTEN/AKT/mTOR signaling pathway and risk of cancer. However, some limitations need to be addressed in this meta-analysis. First, only Asian population was involved, lack of the samples of other ethnicities such as Caucasian, African in this study, thus a wider spectrum of subjects should be conducted on various ethnicities in the future. Second, only 3 studies were included for the SNP rs2494752, therefore considering the limited small size of rs2494752, we cannot rule out the possibility that the results may be by chance, although the number of study participants met the requirement for analysis. Third, all studies included in the present systematic review were reported in Chinese or English, yet other languages publications may include the relevant studies, which may be the main source of publication bias in our meta-analysis. Finally, only five SNPs located in the 3′UTR or 5′UTR of the candidate genes were chosen, however, the SNPs of exon or intron region was not included. Thus, the limited SNPs were not sufficient to capture most genetic information of pTEN/AKT/mTOR signaling pathway, more SNPs should be included in the study, and interaction with others genes should be investigated in the future updated meta-analysis.

In conclusion, this meta-analysis was performed included the latest publications, and provided a more precise prevalence estimate for associations of five SNPs of pTEN/AK/mTOR pathway with the risk of cancer. We found that *mTOR* rs2295080 TG, GG genotype and GG/TG genotype carriers showed an decreased in the overall cancer risk, urinary system cancer and digestive system cancer, nevertheless TT genotype of rs2295080 was associated with increased the risk of blood system cancer. Carrying rs701848 CC,TC genotype and CC/TC genotype were associated with an increased overall cancer risk, especially in digestive system cancer and urinary system cancer. Moreover, a significant increased association was observed between rs2494752 AG and GG/AG genotype and overall cancer risk. Therefore, this present study provides the most valid cancer prevalence estimate to date for Asian population, which is a necessary foundational piece for further research on this topic.

### Novelty and Impact Statements

In this meta-analysis of Asian population, the carriers of *mTOR* rs2295080 TG, GG genotype and GG/TG genotypes showed a significantly decreased the risk of overall cancer, the urinary system cancer and digestive system cancer. Furthermore, the SNP rs701848 CC, TC genotype and CC/TC genotype of *pTEN* were observed increased susceptibility of overall cancer and the subgroup of the urinary and digestive cancer. Moreover, carrying *AKT1* rs2494752 AG and GG/AG genotypes showed an increased overall cancer risk.

## Electronic supplementary material

supplementary materials

## References

[1] Siegel RL, Miller KD, Jemal A (2015). Cancer statistics 2015. CA Cancer J Clin.

[2] Chen W (2016). Cancer statistics in China, 2015. CA Cancer J Clin.

[3] Lim HJ, Crowe P, Yang JL (2015). Current clinical regulation of PI3K/PTEN/Akt/ mTOR signaling in treatment of human cancer. J Cancer Res Clin Oncol.

[4] McCubrey JA (2012). Mutations and deregulation of Ras/Raf/MEK/ERK and PI3K/PTEN/Akt/mTOR cascades which alter therapy response. Oncotarget.

[5] Johnson SM (2010). Novel expression patterns of PI3K/Akt/mTOR signaling pathway components in colorectal cancer. Journal of the American College of Surgeons.

[6] Xu M (2013). A polymorphism (rs2295080) in *mTOR* promoter region and its association with gastric cancer in a Chinese population. PLOS One.

[7] Cao Q (2012). A functional variant in the *MTOR* promoter modulates its expression and is associated with renal cell cancer risk. PloS One.

[8] Xu M (2015). Functional promoter rs2295080 T > G variant in *mTOR* gene is associated with risk of colorectal cancer in a Chinese population. Biomed Pharmacother.

[9] Yuan T, Cantley L (2008). PI3K pathway alterations in cancer: variations on a theme. Oncogene.

[10] Zoncu R, Efeyan A, Sabatini DM (2011). mTOR: from growth signal integration to cancer, diabetes and ageing. Nature reviews Molecular cell biology.

[11] Fingar DC (2004). mTOR controls cell cycle progression through its cell growth effectors S6K1 and 4E-BP1/eukaryotic translation initiation factor 4E. Molecular and cellular biology.

[12] Laplante M, Sabatini DM (2012). mTOR signaling in growth control and disease. Cell.

[13] Edinger AL, Thompson CB (2002). Akt maintains cell size and survival by increasing mTOR-dependent nutrient uptake. Molecular biology of the cell.

[14] Chung JY (2009). The expression of phospho-AKT, phospho-mTOR, and PTEN in extrahepatic cholangiocarcinoma. Clinical cancer research.

[15] Chen J (2012). Genetic variations in a *PTEN/AKT/mTOR* axis and prostate cancer risk in a Chinese population. PLoS One.

[16] Huang L (2012). Association of genetic variations in *mTOR* with risk of childhood acute lymphoblastic leukemia in a Chinese population. Leuk Lymphoma.

[17] Li Q (2013). Polymorphisms in the *mTOR* gene and risk of sporadic prostate cancer in an Eastern Chinese population. PloS One.

[18] Zhu ML (2013). Polymorphisms in *mTORC1* genes modulate risk of esophageal squamous cell carcinoma in eastern Chinese populations. J Thorac Oncol.

[19] Wang MY (2015). Genetic variations in the *mTOR* gene contribute toward gastric adenocarcinoma susceptibility in an Eastern Chinese population. Pharmacogenet Genomics.

[20] Zhao P (2013). Analysis of polymorphism of *mTOR* gene in children with leukemia. J Clin Pediatr.

[21] Zhu J (2015). Associations of *PI3KR1* and *mTOR* polymorphisms with esophageal squamous cell carcinoma risk and gene-environment interactions in Eastern Chinese populations. Sci Rep.

[22] Zhao Y (2016). Impacts of the *mTOR* gene polymorphisms rs2536 and rs2295080 on breast cancer risk in the Chinese population. Oncotarget.

[23] Zhang J (2015). Polymorphism in the *mTOR* promoter is associated with risk of renal cell carcinoma. J Mod Urol.

[24] Fallah S, Korani M, Hajimirza M, Seifi M (2015). Association between genetic variants of *Akt1* and Endometrial Cancer. Biochem Genet.

[25] Wang MY (2016). A Functional Polymorphism (rs2494752) in the *AKT1* Promoter Region and Gastric Adenocarcinoma Risk in an Eastern Chinese Population. Sci Rep.

[26] Zhu J (2016). Polymorphisms in the *AKT1* and *AKT2* genes and oesophageal squamous cell carcinoma risk in an Eastern Chinese population. J Cell Mol Med.

[27] Tang, Q. S. The genetic polymorphisms of PTEN/PI3K/AKT signaling pathway are associated with the susceptibility and neoadjuvant chemotherapy response and prognosis of breast cancer (Doctoral Thesis). China Medical University, Shenyang, Liaoning, China (2014).

[28] Mao, L. Q. Association of *mTOR* polymorphisms and genetic susceptibility of hepatocellular carcinoma (Master’s Thesis). Guangxi Medical University, Xining, Guangxi, China (2013).

[29] He J (2013). Genetic variations of *mTORC1* genes and risk of gastric cancer in an Eastern Chinese population. Mol Carcinog.

[30] Liu YC (2014). Association of *mTOR* polymorphisms with risk of hepatocellular carcinoma. Chin J Public Health.

[31] Zhou, J. F. Research of association of single nucleotide polymorphisms of PTEN gene with china laryngeal cancer (Master’s Thesis). Jilin University, Jilin, Changchun, China (2006).

[32] Zhai, Y. Study on the correlationgship of *PTEN* gene and laryngeal carcinoma (Master’s Thesis). Jilin University, Changchun, Jilin, China (2009).

[33] Liu B, Liu Y, Zhou JF, Long HZ, Zhu W (2008). Correlation analysis of *PTEN* gene single nucleotide polymorphism in laryngocarcinoma. Chin J Cancer Prev Treat.

[34] Song ZX, Zhu W, Li P, Liu B (2009). Association between anti-oncogene PTEN single nucleotide polymorphism and laryngocarcinoma. Journal of Jilin University (Medicine Edition).

[35] Shi GL, Qin HY, Chen Q (2009). The association between lung cancer and the single nucleotide polymorphisms of *PTEN* gene. Modern. Oncology.

[36] Ding, J. The association of *PTEN* gene with the development of Hepatocellular Carcinoma (Master’s Thesis). Suzhou University, Suzhou, Jiangsu, China (2011).

[37] Liu N, Xu C (2015). Association of *MDM2* and *PTEN* gene polymorphisms and susceptibility of esophageal squamous carcinoma in Anyang area. Chin J Digest Med Imageol (Electronic Edition).

[38] Jang Y (2013). Genetic polymorphisms of *CCND1* and *PTEN* in progression of esophageal squamous carcinoma. Genet Mol Res.

[39] Xu, X., Chen, G., Wu, L. & Liu, L. Association of genetic polymorphisms in *PTEN* and additional gene-gene interaction with risk of esophageal squamous cell carcinoma in Chinese Han population. Dis Esophagus (2015). 10.1111/dote.12428 26541596

[40] Lin L, Zhang Z, Zhang W, Wang L, Wang J (2015). Roles of genetic variants in the PI3K/PTEN pathways in susceptibility to colorectal carcinoma and clinical outcomes treated with FOLFOX regimen. Int J Clin Exp Pathol.

[41] Zhang, Y. G. SNPs in micro RNA Binding Site of EGFR Signaling Pathway and Susceptibility to ESCC. (Master’s Thesis). Zhengzhou University, Zhengzhou, Henan, China (2014).

[42] Song ZX, Liu B, Zhao J, Liu J (2009). Analysis of association between *PTEN* gene single nucleotide polymorphism and stomach neoplasms. Journal of Jilin University (Medicine Edition).

[43] Li, Q. Functional genetic variants in the *mTORC1* related genes contribute to prostate cancer susceptibility and clinical outcomes (Doctoral Thesis). Fudan University, Shanghai, China (2014).

[44] Albert V, Hall MN (2015). mTOR signaling in cellular and organismal energetics. Current opinion in cell biology.

[45] Polivka J, Janku F (2014). Molecular targets for cancer therapy in the PI3K/AKT/ mTOR pathway. Pharmacology & therapeutics.

[46] Shao J (2014). Association of mTOR polymorphisms with cancer risk and clinical outcomes: a meta-analysis. PLoS One.

[47] Zining J, Lu X, Caiyun H, Yuan Y (2016). Genetic polymorphisms of *mTOR* and cancer risk: a systematic review and updated meta-analysis. Oncotarget.

[48] Downes CP (2001). Antagonism of PI3-kinase-dependent signaling pathways by the tumour suppressor protein, PTEN. Biochemical Society Transactions.

[49] Hildebrandt MA (2009). Genetic variations in the PI3K/PTEN/AKT/mTOR pathway are associated with clinical outcomes in esophageal cancer patients treated with chemoradiotherapy. J Clin Oncol.

[50] Chen M (2009). Genetic variations in PI3K-AKT-mTOR pathway and bladder cancer risk. Carcinogenesis.

