# Supplementary material for: Associations of genetic polymorphisms in pTEN/AKT/mTOR signaling pathway genes with cancer risk: A meta-analysis in Asian population
Source: Sci Rep. 2017 Dec 19;7:17844. doi: 10.1038/s41598-017-17250-z (PMC5736732; doi:10.1038/s41598-017-17250-z)
Supplement: Supplementary file 1 — supplementary materials [file 41598_2017_17250_MOESM1_ESM.pdf]

Supplemental Information (SI)

Associations of genetic polymorphisms in pTEN/AKT/mTOR signaling pathway genes with cancer risk：A meta-analysis in Asian population

Zhen Zhang,Qiuchen Chen, Jing Zhang, Yilin Wang, Xiaoyun Hu, Sainan Yin, Miao He, Shu Guan, Wenyan Qin, Qinghuan Xiao, Haishan Zhao, Weifan Yao, Huizhe Wu, and Minjie Wei

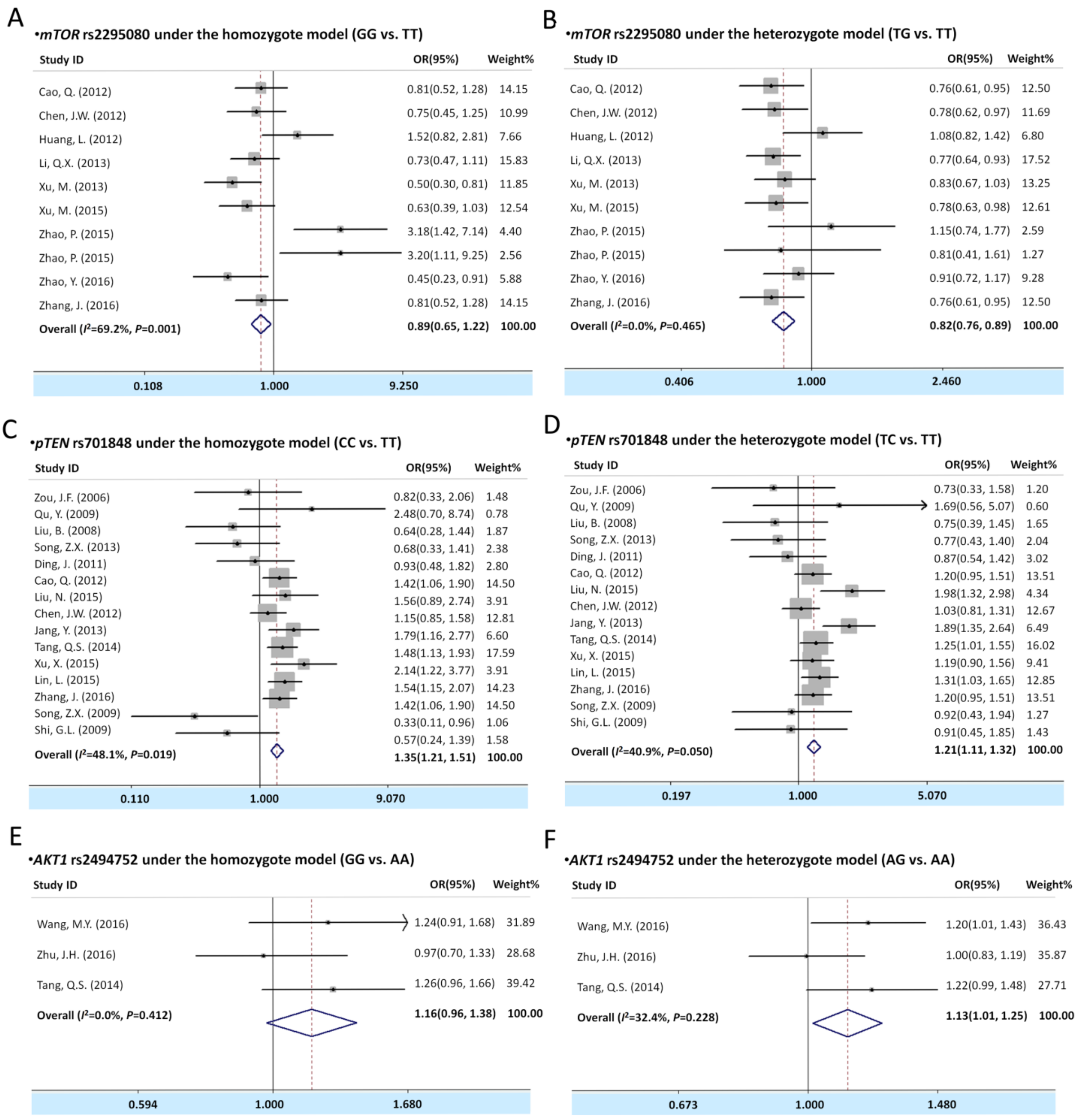

Figure S1. Forest plots of cancer risk with polymorphism of *mTOR* rs2529080 (A,B), *pTEN* rs701848 (C,D) and *AKT1* rs2494752 (E,F) assessing by overall analysis under the homozygoute model (A,C,E), heterozygote model (B,D,F).The estimates of OR(95%CIs) are plotted with a box and a horizontal line for each study.◇, pooled ORs (95% CIs).

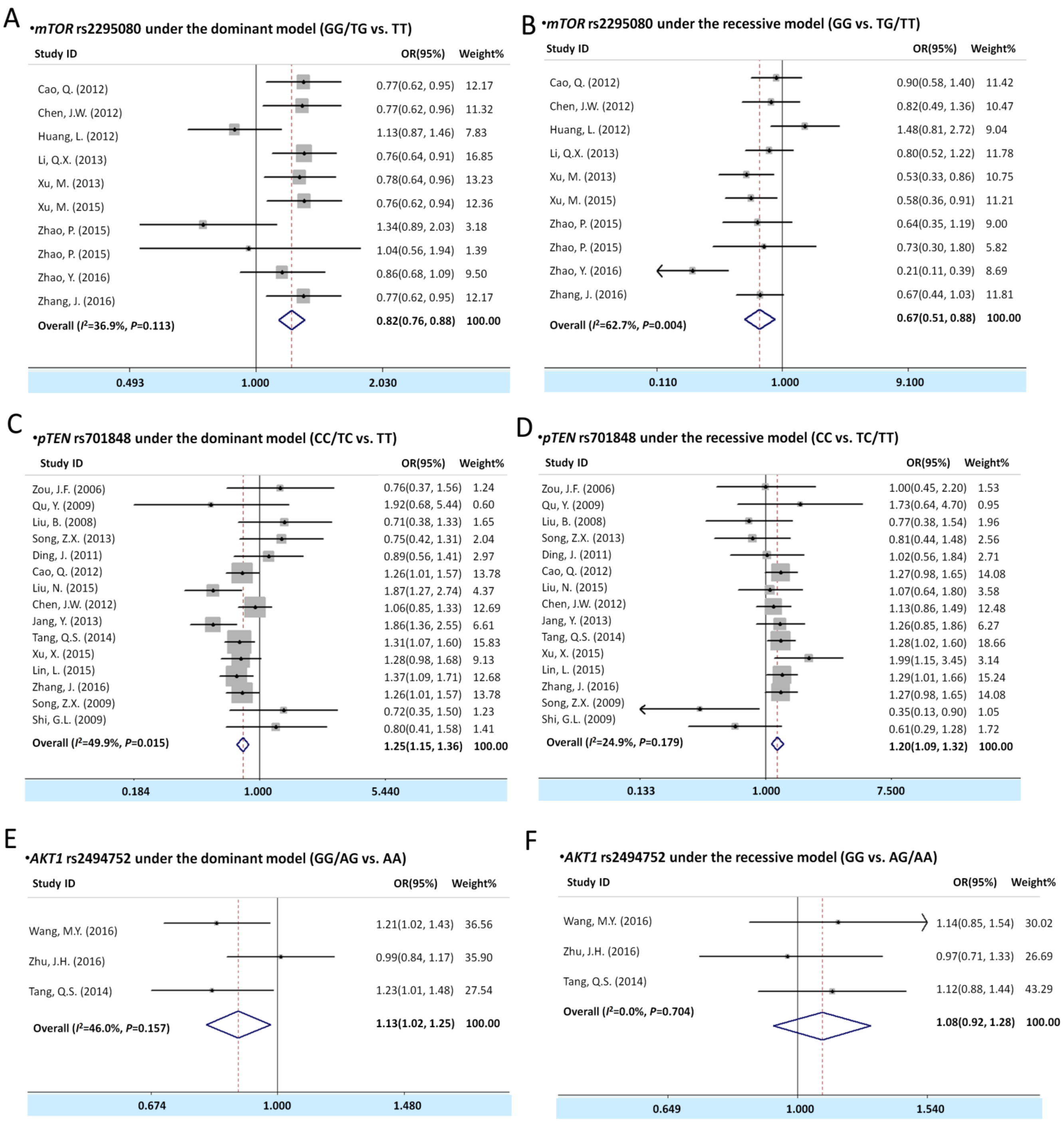

Figure S2. Forest plots of cancer risk with polymorphism of *mTOR* rs2529080 (A,B), *pTEN* rs701848 (C,D) and *AKT1* rs2494752 (E,F) assessing by overall analysis under the dominant model (A,C,E) and recessive model (B,D,F). The estimates of OR(95%CIs) are plotted with a box and a horizontal line for each study. ◇, pooled ORs (95% CIs).

Table S1. Meta-analysis of the association between genetic polymorphisms of PTEN/AKT/mTOR pathway and cancer risk

**Variables**	**No. of cases/ controls**	P^{z*}	**Homozygous OR (95%CI)**	$P^{het\#}$	$I^{2\#}$ **(%)**	P^{z*}	**Heterozygous OR (95%CI)**	$P^{het\#}$	$I^{2\#}$ **(%)**
***mTOR* rs2295080**			**GG vs. TT**				**TG vs. TT**		
Urinary system cancer†	4203/4392	0.070	0.832(0.681,1.015)	0.787	0.0	**0.030**	**0.832(0.705,0.982)**	0.011	69.4
Blood system cancer§	5971/1146	**0.003**	**2.250(1.327,3.815)**	0.264	24.8	0.574	1.065(0.855,1.327)	0.691	0.0
Digestive system cancer ¶	3605/3747	0.169	0.777(0.542,1.113)	0.059	59.6	0.785	0.972(0.795,1.189)	0.005	76.3
**Overall**	8965/9868	0.449	0.913(0.722,1.155)	0.001	62.6	0.104	0.915(0.821,1.019)	0.001	64.5
***pTEN* rs701848**			**CC vs. TT**				**TC vs. TT**		
Oral cavity cancer¥	331/356	0.452	0.831(0.512,1.347)	0.312	16.0	0.292	0.823(0.572,1.183)	0.603	0.0
Digestive system cancer	2418/2662	0.387	1.191(0.802,1.769)	0.000	77.6	0.108	1.235(0.955,1.598)	0.001	73.3
Urinary system cancer	2086/2228	**0.001**	**1.331(1.120,1.582)**	0.561	0.0	**0.049**	**1.145(1.000,1.310)**	0.593	0.0
**Overall**	5882/6284	0.098	1.180(0.970，1.435)	0.000	62.6	**0.030**	**1.154(1.014,1.313)**	0.010	51.1

* P^{z}: the significance of the pooled OR was determined by Z-test, and $P<0.05$ was considered as statistically significant.
# P^{het} and I^2 were calculated by Chi square-based Q-test.
†Urinary system cancer: renal cancer, prostate cancer; § Blood system cancer: acute lymphocytic leukemia, acute myeloid leukemia; ¶Digestive system cancer: gastric cancer, ESCC, hepatocellular cancer, colorectal cancer; ¥Oral cavity cancer: laryngo cancer; $Reproductive system cancer: endometrial cancer.

Table S2. Meta-analysis of the association between genetic polymorphisms of PTEN/AKT/mTOR pathway and cancer risk by recessive and dominant models

**Variables**	***P*z***	**Recessive OR (95%CI)**	***P*het#**	***I*2# (%)**	***P*z***	**Dominant OR (95%CI)**	***P*het#**	***I*2# (%)**
***mTOR* rs2295080**		**GG/TG vs. TT**				**GG vs. TG/TT**		
Urinary system cancer†	0.225	0.885(0.727,1.078)	0.953	0.0	0.118	0.699(0.446,1.095)	0.000	96.3
Blood system cancer§	**0.004**	**2.253(1.300,3.904)**	0.227	32.6	0.142	1.169(0.949,1.440)	0.722	0.0
Digestive system cancer ¶	0.111	0.788(0.588,1.056)	0.168	40.6	**0.014**	**0.491(0.279,0.865)**	0.000	97.6
**Overall**	0.574	0.939(0.756,1.168)	0.005	57.7	0.020	0.707(0.527,0.948)	0.000	96.1
***pTEN* rs701848**		**CC/ TC vs. TT**				**CC vs. TC/TT**		
Oral cavity cancer¥	0.674	0.924(0.639,1.336)	0.568	0.0	0.250	0.818(0.581,1.152)	0.409	0.0
Digestive system cancer	0.195	1.106(0.950,1.288)	0.008	65.7	0.160	1.215(0.926,1.594)	0.000	78.4
Urinary system cancer	**0.008**	**1.229(1.054,1.432)**	0.784	0.0	**0.006**	**1.195(1.053,1.357)**	0.476	0.0
**Overall**	**0.003**	**1.154(1.051,1.267)**	0.039	42.2	**0.042**	**1.156(1.005,1.328)**	0.001	62.0

*Pz: the significance of the pooled OR was determined by Z-test, and $P<0.05$ was considered as statistically significant.

# Phet and I2 were calculated by Chi square-based Q-test.

†Urinary system cancer: renal cancer, prostate cancer; § Blood system cancer: acute lymphocytic leukemia, acute myeloid leukemia; ¶Digestive system cancer: gastric cancer, ESCC, hepatocellular cancer, colorectal cancer; ¥Oral cavity cancer: laryngo cancer; $Reproductive system cancer: endometrial cancer.
